# An ion treatment planning framework for inclusion of nanodosimetric ionization detail through cluster dose

**DOI:** 10.1002/mp.70579

**Published:** 2026-07-21

**Authors:** Simona Facchiano, Ramon Ortiz, Remo Cristoforetti, Naoki D‐Kondo, Oliver Jäkel, Bruce Faddegon, Niklas Wahl

**Affiliations:** ^1^ Department of Medical Physics in Radiation Oncology German Cancer Research Center (DKFZ) Heidelberg Germany; ^2^ National Center for Radiation Research in Oncology (NCRO) Heidelberg Institute for Radiation Oncology (HIRO) Heidelberg Germany; ^3^ Faculty of Physics and Astronomy Heidelberg University Heidelberg Germany; ^4^ Department of Radiation Oncology University of California San Francisco (UCSF) San Francisco California USA; ^5^ Department of Radiation Oncology Heidelberg Ion Beam Therapy Center (HIT) University Hospital Heidelberg (UKHD) Heidelberg Germany

**Keywords:** carbon ions, cluster dose, helium, ionization parameter, Monte Carlo simulation, nanodosimetry, pencil‐beam algorithm, planning, protons, radiotherapy treatment planning

## Abstract

**Background:**

Nanodosimetry relates the cumulative or statistical moments of Ionization Detail (ID) with biological endpoints of relevance to cancer radiotherapy using charged particles. This association suggests to develop an additional physics‐detailed layer of modeling that may complement biological modeling and treatment planning. The recently introduced *cluster dose* $g^{\left(I_{p}\right)}$ may serve as a purely physical quantity bridging the *Ionization Parameter* ($I_{p}$) to the macroscopic treatment planning scale.

**Purpose:**

In this work, we developed a framework to enable flexible and direct cluster dose optimization using a pencil‐beam algorithm, which we validated with condensed history Monte Carlo (MC) simulations.

**Methods:**

Cluster dose combines the contributions to $I_{p}$ from all particles within a macroscopic volume. Our framework, implemented in the open source planning toolkit matRad, utilizes the particle and energy‐dependent $I_{p}$ values from an ID database precomputed from MC track strucure (MCTS) simulations. First, we create pencil‐beam (PB) kernels, including fluence spectra, from condensed history MC simulations. For a water box phantom and a representative prostate patient, we create treatment plans optimized on dose and cluster dose $F_{5}$ coverage and homogeneity for protons, helium and carbon ions. Plans were validated with Geant4/TOPAS MC.

**Results:**

Our framework provided accurate, practical cluster dose calculation and planning. PB algorithms achieve typical accuracy for cluster dose calculation comparable to dose calculation. Recalculation with TOPAS on the box phantom yielded 3D gamma passing rates (GPRs) greater than 97%. For the prostate patient, GPRs exceeded 98%. Both used the 3%/3mm criterion with a threshold of 10% of the maximum dose. Using cluster dose $F_{5}$ optimization, homogeneous cluster dose target coverage was achieved in all plans. A constant cluster dose prescription across all ion species shows the expected decrease in required absorbed dose for heavier ions.

**Conclusions:**

We demonstrate that fast, direct cluster dose calculation and optimization is feasible using MC validated planning with PB algorithms. Cluster dose prescription and optimization results in the expected cluster dose coverage and physical dose levels depending on the respective primary ion.

## 1 INTRODUCTION

Current radiotherapy treatment planning for protons and heavier ions relies on relative biological effectiveness (RBE) models, which translate the absorbed dose into the isoeffective reference dose. For proton therapy, a constant RBE of 1.1 is employed in clinical routines, despite evidence of variations with depth, biological endpoint, and patient‐specific factors^1^, ^2^, ^3^. For carbon ions, different RBE models are used for clinical purposes, such as the local effect model (LEM)^4^, ^5^, ^6^ and the microdosimetric kinetic model (MKM),^7^, ^8^, ^9^ leading to deviations in prescribed absorbed dose of up to 15%.^10^ In order to improve the biological effect in the target and healthy tissue sparing, LET has been proposed as a quantity to steer directly in RTP. But while it is possible to reduce LET in healthy tissues without substantially compromizing the dose distribution,^11^, ^12^, ^13^ the nonlinear behavior observed in multiple field treatment planning indicates that LET is not a direct predictor of biological effect.^14^ This motivates the incorporation of additional physical quantities into RTP to establish a stronger link between ion track properties and biological response. Such quantities may, for example, be derived from microdosimetry or nanodosimetry.

Nanodosimetry has been gaining interest since the 1970s^15^ with the subsequent development of experimental nanodosimetry and MCTS simulations. Through nanodosimetry, the link has been demonstrated between the distribution of energy depositions within nanometric volumes surrounding a particle track – resulting in ionizations and excitations – with complex DNA damage, such as double‐strand breaks, and repairability.^16^

More recently, it has been suggested to use *Ionization Detail* (ID) to condense nanodosimetric track‐structure information in useful quantities. ID is defined as the spatial distribution of ionization events within nanometer sized regions surrounding a particle track,^17^ and characterized by the *frequency ionization cluster size distribution* (fICSD). The fICSD information can be condensed into statistical moments, or cumulative quantities known as *Ionization parameters* ($I_{p}$).^17^, ^18^ Studies have confirmed the correlation between such cumulative probabilities and inactivation cross sections in mammalian cells.^19^, ^20^, ^21^, ^22^, ^23^

Therefore, the use of ID enables translation from the nanoscopic to the macroscopic scale. When successful, this enables direct treatment planning on ID statistical moments. For example, Burigo et al.^24^ proposed to minimize the variance of cluster frequency in addition to common RBE‐based planning.

Recently, Faddegon et al.^18^ proposed a generalized paradigm for ID‐based RTP. They presented a mathematical model to bridge ID and its $I_{p}$ to the macroscopic RTP scales through the definition of a macroscopic (voxel‐averaged) $I_{p}$ and the novel concept of *cluster dose*, indicated with $g^{\left(I_{p}\right)}$. This *cluster dose* can be understood as a fluence‐dependent physical quantity “counting” the number of ionization clusters per unit mass (with the specific cluster sizes defined by the $I_{p}$), aggregating the contribution of primary and secondary particles. Formally, cluster dose can operate as a dose surrogate, quantifying ionization clusters per unit mass instead of absorbed energy. This may be analogized as “LET relates to dose as $I_{p}$ relates to cluster dose.”

For the cell lines and radiation modalities tested, their results indicate that the association between some of the physical quantities defined within the model and cell survival appears to be particle‐independent, although further studies are needed to confirm this more broadly. For example, for $I_{p}$ defined as $F_{k}$, the number of clusters of *k* or more ionizations in a nanometer‐sized volume of specific dimensions, $F_{5}$ was preferred for aerobic cells (showing the closest association between cell survival and $F_{k}$ for different types of charged particles of the same fluence), while $F_{7}$ was preferred for hypoxic cells. Preliminary data suggested that for the preferred $I_{p}$, defined for its association with cell survival, the same cluster dose level is expected to lead to similar levels of cell survival.

This initial evidence warrants to investigate into the inclusion of the proposed cluster dose and $I_{p}$ in treatment planning. Cluster dose, as a physical quantity that is potentially particle‐agnostic, could be directly prescribed in RTP as a constraint on RBE‐weighted absorbed dose to reduce biological variability reported in the literature^25^, ^26^ with respect to other physical quantities such as LET, or even obviate RBE models in optimization.

The inclusion of cluster dose can be facilitated by MCTS databases of nanodosimetric quantities to be employed in condensed history MC in order to directly calculate averaged or weighted quantities on the treatment planning scales.^17^ Nevertheless, calculation times of MC simulations for charged particles are often prohibitive to application in optimization, especially for inclusion of nontrivial quantities, although more effort has been invested into fast GPU‐based MC methods.^27^, ^28^, ^29^

Introducing optmization strategies for fast nanodosimetric treatment planning would be highly valuable for the field, as it would support future validation of methodological choices and assessment of uncertainties stemming from track‐structure simulations.

In this context, the purpose of this paper is twofold: (1) to introduce and validate fast cluster dose calculation using PB algorithms consistent with MC calculation. (2) To introduce and demonstrate feasibility of a generalized plan optimization approach using cluster dose prescriptions. We have implemented our framework for cluster dose calculation and optimization in the open source planning toolkit matRad.^30^ The cluster dose calculation and optimization algorithms are outlined for multiple definitions of $I_{p}$ across three ion species: protons, helium, and carbon ions. Plans are generated on phantom geometries as well as a prostate patient, relying on dose and cluster dose prescriptions. All plans and corresponding cluster dose calculations are validated with Geant4 condensed history MC^31^ in TOPAS^32^, ^33^ using dose differences and γ‐analyses.^34^

## 2 MATERIALS AND METHODS

### 2.1 From ID to cluster dose: An overview of the mathematical model

Faddegon et al.^18^ introduced a comprehensive formalism bridging the nanoscopic $I_{p}$ to the macroscopic world, using “cluster dose” as a fluence‐dependent target quantity for treatment planning. We adopt this specific formalism throughout this study.

The formalism relies on the previously introduced concepts of *ionization cluster size* ν, and of fICSD, which together describe the spatial distribution of ionizations along a particle track.^17^, ^35^, ^36^, ^37^ The frequency distribution $f^{c}\left(\nu \right)$ is defined as the absolute number of ionization clusters of size ν per unit path length formed along the track of a particle of class *c*, which means their type and energy.

The size ν of a cluster was determined for the purposes of this study by counting the ionizations within a nanometer‐sized region comparable to a DNA segment of 10 base pairs, such that two strand breaks occurring within the same volume can cause complex DNA damage, for example a Double Strand Break (DSB).^38^, ^39^, ^40^, ^41^

The $I_{p}^{c}$ reflects the information of the fICSD collapsed into a single scalar for the respective particle class c. While the computation of the $I_{p}$ has been formalized as the action of a linear operator on f, two types of $I_{p}$ were essentially highlighted and will be used in this paper:

(1) $$F_{k}=\sum_{\nu =k}^{\infty }f^{c}\left(\nu \right)\;\;N_{k}=\sum_{\nu =k}^{\infty }\nu f^{c}\left(\nu \right)\;.$$

Due to $f^{c}\left(\nu \right)$ representing the absolute cluster frequency, it follows that $F_{k}$ is the cumulative frequency, that is, the number of clusters with k or more ionizations produced by a particle class and per unit path length. Similarly, $N_{k}$ is the sum of cluster size weighted by the correspondent absolute frequency for clusters with size ν≥k. Thus it represents the absolute number of ionizations contained in the clusters with a minimum size k, produced by a particle of class c, per unit path length.

Usage of these $I_{p}$ on a macroscopic level is now motivated with the assumption that particles of different type and energy, which produce a similar clustering of ionization events on the nanometer scale, are expected to lead to a similar complexity in the local DNA damage.^20^ If the clustering properties can be translated to aggregated or averaged macroscopic quantities, such as *cluster dose* and *track‐averaged* $I_{p}$, a physical measure linking to biological isoeffectiveness across particle species could be provided to the planner.

*Cluster dose* $g_{j}^{\left(I_{p}\right)}$ is the macroscopic generalization of the $I_{p}$. It is the fluence‐weighted sum of $I_{p}^{c}$ from all the particle classes within a macroscopic voxel,

(2) $$g_{j}^{\left(I_{p}\right)}=\frac{1}{\rho_{0}}\sum_{c\in C_{j}}\phi_{j}^{c}I_{p}^{c}=\frac{1}{\rho_{0}}\sum_{c\in C_{j}}\frac{t_{j}^{c}}{V_{j}}I_{p}^{c}\;,$$

where, $C_{j}$ is the set of particle classes within voxel j, $\phi_{j}^{c}$ indicates the fluence of particle class c within voxel j, $I_{p}$ can be $N_{k}$ or $F_{k}$, $t_{j}^{c}$ is the track length of particle class c within voxel j, $V_{j}$ the voxel volume, and $\rho_{0}$ is the density of the medium used to compute the database (i.e., water). Analogous to absorbed dose, representing the deposited energy per unit mass, cluster dose represents the number of ionizations, or ionization clusters, per unit mass. It is therefore expressed in units of inverse mass. Cluster dose is fluence dependent and therefore suitable for direct optimization.

Equation 2 also highlights the advantage of using absolute frequency distributions in this formalism instead of relative frequencies or empirical probability distributions. Unlike ICSD probabilities, the fICSD is not bounded between 0 and 1. Instead, the fICSD is normalized to the number of primary particles and to the track length. This definition of fICSD yields the average number of clusters of a given size ν along the particle track c, providing increased statistics, reduced computational cost, and direct applicability within condensed‐history MC simulations.

Using absolute frequencies in the computation of $I_{p}$ results in $I_{p}$ representing absolute cluster frequencies or ionization counts. Weighting these frequencies, defined per particle and unit track length, with the scored track length (or fluence) in condensed‐history MC simulations therefore leads to the accumulation of absolute numbers of ionization clusters. Both the fICSD and $I_{p}$ are normalized to the track length, which means that the dependence on the number of sensitive volumes is removed. In other words, doubling the length of the track segment within the scoring region also doubles, on average, the number of sensitive volumes scoring a cluster of a given size ν.

This formalism facilitates the calculation of cluster dose from fluence (or track length) without requiring additional normalization to the total number of clusters for a given particle class c.

It is also possible to define a voxel‐averaged $I_{p}$ computed as the fluence‐averaged $I_{p}$ within a voxel:

(3) $$I_{p}^{C_{j}}=\frac{\sum_{c\in C_{j}}\phi_{j}^{c}I_{p}^{c}}{\sum_{c\in C_{j}}\phi_{j}^{c}}$$

from which it follows the relation between cluster dose, $I_{p}$ and fluence:

(4) $$g_{j}^{\left(I_{p}\right)}=\frac{1}{\rho }\phi_{j}I_{p}^{C_{j}},\;\mathrm{with}\;\phi_{j}=\sum_{c\in C_{j}}\phi_{j}^{c}\;.$$

Equation 4 shows that if $g^{\left(I_{p}\right)}$ is analogous to dose, the averaged $I_{p}^{C_{j}}$ corresponds to stopping power and can be interpreted as a radiation quality metric. Consequently, introducing cluster dose optimization alongside the steering of the $I_{p}$ becomes essential for managing radiation quality effectively.

### 2.2 Practical cluster dose calculation

The MC method is currently regarded as one of the most accurate approaches for simulating particle transport in a medium, as its stochastic nature reflects the underlying physics of particle interactions. Most often, radiotherapy uses condensed history MC, which groups events to enhance computational efficiency while retaining accuracy relevant to treatment planning.^42^, ^43^, ^44^, ^45^ Thus, MC methods would be the primary choice for nanodosimetric calculations, yet they are rarely fully integrated into the ion planning process, especially for ions treatments. Instead, they are often used for secondary dose verification or for research applications.^46^, ^47^, ^48^, ^49^, ^50^

Ion therapy treatment planning usually utilizes PB algorithms (often derivatives from Ref. 51) interpolating from a precomputed kernel database created from MC simulations or measurements in water. Simplifying the inclusion of lateral heterogeneities and relying on water‐equivalent path length, they provide the speed necessary for dose influence matrix calculations at reduced accuracy.^44^, ^49^ The dose influence matrix is then used to map individual beamlet fluences to the full dose distribution for changing input fluence, which is primarily necessary during iterative plan optimization.

Building on this principle, we investigated two approaches for nanodosimetric cluster dose calculation – a scoring method embedded into MC simulation for forward (cluster) dose calculations as well as a PB kernel algorithm for inverse planning via the calculation of cluster dose influence matrices.

Section 2.1 shows that the required inputs for cluster dose calculation are the fICSD (or the $I_{p}$), and the primary and secondary particle fluence. The former can be provided as a precomputed ID database, with the general format outlined below (see *ID Database*). Particle fluence can be obtained through MC, from which cluster dose can be directly calculated within a simulation. Alternatively, fluence can be stored as fluence kernels to calculate cluster dose in post‐processing and use with PB algorithm.

#### 2.2.1 ID Database

The cluster dose calculation methods described in the following sections both rely on the availability of a precomputed database of ID, which contains the particle and energy‐dependent fICSD (or $I_{p}$). The general format we describe here is inspired by the MCTS database that we use. For each particle type p, an fICSD look up table is required – or equivalently, two look up tables: one for $F_{K}$ and one for $N_{K}$. The fICSD is tabulated as a function of ν and *E*, while $F_{k}$ and $N_{k}$ are tabulated as a function of k and *E*. Given this format, the fICSD of a proton with kinetic energy *E*, that is $f^{c}\left(\nu \right)$ with c={proton,E} (see Section 2.1), is obtained by linear interpolation of the proton fICSD table, and analogously for the $I_{p}$, that is $F_{k}$ and $N_{k}$.

The MCTS database was generated using as scoring region a $100\times 100\times 100\;nm^{3}$ cube of water, in which 53 621 smaller cylinders of 3.4nm height and 2.3nm diameter were placed with same orientation, tight packing and no overlap, in order to maximize the number of sampling volumes within the scoring region. This geometry was presented by Ortiz et al.^52^, while previous work from Ramos–Méndez et al.^17^, ^18^ used a different scoring geometry. The radiation source was randomly placed within a sphere of 34nm diameter, made of vacuum, and positioned in the center of the scoring region. No sampling volumes were included within the source sphere. The source particles were generated within the source sphere with random placement and direction, and the ionization frequencies produced in the scoring region were divided by the number of primary particles and path length. For further details, we refer the reader to Ortiz et al. ^52^

#### 2.2.2 Computing cluster dose g with MC

Given a precomputed *ID database* with the format described, cluster dose can be directly scored within a MC simulation. According to Equation 2, cluster dose is proportional to the track‐weighted sum of the $I_{p}$ of the particle classes within a voxel. Thus it is sufficient to cumulate the product between the particle track length and the $I_{p}$, the latter being interpolated on the fly using the current particle type and energy assigned to the step. This method is considered to provide the gold‐standard reference value, as MC codes have previously been validated for dose calculation.^31^, ^48^, ^53^, ^54^ Additionally, the $I_{p}$ (or the frequencies fICSD) are interpolated for each single scored particle step.

#### 2.2.3 Pencil‐beam algorithm for cluster dose g calculation

The kernel database used for dose calculation using PB algorithm generally contains the depth‐dose profile, representing the integrated dose at each depth, as well as the lateral beam broadening due to Multiple Coulomb Scattering. The latter is often modeled using Gaussian distributions (single, double, or multiple), with the width(s) of the distribution varying as a function of depth. Thus we extend the kernel database in order to be able to calculate the PB cluster dose.

We propose two methods for PB cluster dose calculation – which are further explained below and indicated as *Flexible* and *Fast* methods: (1) using particle spectra and directly interpolating the $I_{p}$ from the ID database, and (2) using a precomputed cluster‐dose kernel for the respective $I_{p}$. Thus we extend our database by including both precomputed fluence and cluster dose kernels obtained through MC simulations (see Section 2.2.2).

For the fluence, within a single pencil‐beam and for each relevant primary and secondary particle type – identified by the atomic number *Z* and, if applicable, the atomic mass *A* – we store: the fluence as function of depth $\phi^{\left[Z,A\right]}\left(d\right)$, fluence as function of the energy bin and depth $\phi^{\left[Z,A\right]}\left(E_{\text{bin}},d\right)$, and the lateral beam broadening as function of depth. For the latter we consider a triple radial Gaussian model (for details, see Section A.1).

For cluster dose, we include depth profiles $g^{\left(I_{p}\right)}\left(d\right)$ of PB in the kernel database. This can be achieved either by directly scoring the track‐weighted sum of the $I_{p}$ in each voxel (voxel‐averaged $I_{p}$) during a MC simulation, as detailed in Section 2.2.2, or through post‐processing. The latter approach requires the fluence kernel database and Equation 2 to compute the integrated cluster dose at each depth bin. Specifically, directly following Equation 2, the contribution of a particle type s=(Z,A) to the cluster dose in a depth bin, including the full set of particle classes (energies) of that particle type, is expressed as the following matrix product:

(5) $$g^{\left(I_{p}\right)\left[s\right]}\left(d_{j}\right)=\frac{1}{\rho_{0}}\sum_{E_{i}}I_{p}^{\left[s\right]}\left(E_{i}\right)\phi^{\left[s\right]}\left(E_{i},d_{j}\right)$$

where, $\phi^{\left[s\right]}\left(E_{i},d_{j}\right)$ represents the fluence of particle type s, binned by energy and depth, and stored in the database. The depth binning is linear, with finer binning of 0.1mm around the peak, 5mm binning in the plateau region, and 10mm binning in the tail. The energy binning is also linear, with the bin size set to 1 MeV times the atomic mass A of the primary particle. The $I_{p}^{\left[s\right]}\left(E_{i}\right)$ is interpolated from the ID database for energy $E_{i}$. The total cluster dose in depth bin $d_{j}$ is then given by the sum of $g^{\left(I_{p}\right)\left[s\right]}\left(d_{j}\right)$ over all particle types s.

##### Flexible cluster dose calculation with spectral fluence kernels

The first approach relies on precomputed spectral fluence kernels for primary and secondary particles, allowing for on‐the‐fly interpolation of the ID database.

Equation (5) expresses the contribution of a particle type s to cluster dose at a given depth. Its contribution within a bin at a radial distance is then obtained by scaling the depth contribution with the corresponding model of lateral scattering. The total cluster dose within the same bin is then the sum over all the particle types (for details, see the Section A.2).

##### Fast cluster dose calculation from precomputed cluster dose kernels

The second approach targets less computational effort by precomputing cluster dose kernels, forgoing the flexibility of exchanging or modifying the ID database. Total cluster dose depth profiles $g^{\left(I_{p}\right)}\left(d_{j}\right)$ are stored in the kernel database (similarly to dose kernels), accounting for all primary and secondary particles available within the database and contributing to the cluster dose. In this approach, the lateral scattering modeling for the absorbed dose kernels is assumed to approximate the cluster dose lateral broadening (for details, see the Section A.3).

### 2.3 Cluster dose optimization

One of the main goals of this work is to be able to directly optimize with prescriptions on cluster dose. Thus simultaneous optimization of (RBE) dose and cluster dose can be described by the following problem:

(6) $$\begin{array}{cc} & \underset{w}{\mathrm{minimize}\;}\;\chi \left(d,g\right)=\sum_{n=1}^{N}\left(p_{n,d}f_{n}\left(d\right)+p_{n,g}h_{n}\left(g\right)\right)\\ & \mathrm{subject}\;\mathrm{to}\\ & d\in U=\cap_{n}U_{n}\;U_{n}=\left\{d|d^{\min}<d_{i}<d^{\max},\;\forall i\in J_{n}\right\}\\ & g\in V=\cap_{n}V_{n}\;V_{n}=\left\{g|g^{\min}<g_{i}<g^{\max},\;\forall i\in J_{n}\right\}\\ & \;\;d_{i}=\sum_{j}D_{\mathrm{ij}}w_{j}\;\;\forall i\\ & \;\;g_{i}=\sum_{j}G_{\mathrm{ij}}w_{j}\;\;\forall i\\ & \;\;w_{i}\geq 0\;\;\forall i \end{array}$$

where *N* is the number of structures within the RTP geometry, for example, target volumes and OARs. The beamlets fluence weights are indicated by vector w. The associated dose and cluster dose objective functions are indicated as $f_{n}\left(d\right)$ and $h_{n}\left(g\right)$ respectively, with $p_{n,d}$ and $p_{n,g}$ the corresponding penalties. The set of constraints acting on absorbed dose and cluster dose are indicated by U and V. If n is a specific structure (e.g. target, OAR, etc.), $U_{n}$ are the respective dose constraints on structure n, $V_{n}$ the respective cluster dose constraints, and $J_{n}$ the voxel indices.

As cluster dose can now be expressed as a linear transformation of fluence to cluster dose via the cluster dose influence matrix ($G_{\mathrm{ij}}$), we chain dosimetric prescriptions on cluster dose distribution to the fluence analogously to dose, with respective considerations for the derivatives. This enables us to use the same objective and constraint functions as for conventional dose‐based treatment planning, but instead acting on cluster dose. In this work we use three objective functions, that is least‐squares and variance minimization to achieve homogenous cluster dose in targets, and squared overdosing for OARs. A more explicit form for the objective function is:

(7) $$\begin{array}{cc} \chi (d,g) & =\sum_{n=1}^{N_{\text{TARGET}}}p_{n,d}^{T}{\left(d_{i}-d_{n}^{ref}\right)}^{2}\\ & \;+\sum_{n=1}^{N_{\text{OAR}}}p_{n,d}^{O}{\left(d_{i}-d_{n}^{ref}\right)}^{2}\theta \left(d_{i}-d_{n}^{ref}\right)\\ & \;+\sum_{n=1}^{N_{\text{TARGET}}}p_{n,g}^{T}{\left(g_{i}-\bar{g}\right)}^{2}\\ & \;+\sum_{n=1}^{N_{\text{OAR}}}p_{n,g}^{O}{\left(g_{i}-g_{n}^{ref}\right)}^{2}\theta \left(g_{i}-g_{n}^{ref}\right) \end{array}$$

where the first two lines of the Equation represent the objective function relative to dose, and the third and fourth lines are relative to cluster dose. Here, $d_{i}=d_{i}\left(w\right)$ and $g_{i}=g_{i}\left(w\right)$ indicate respectively dose and cluster dose for single voxel i. The target objective contains $\bar{g}$, that is the average cluster dose g(w) over all the target volume.

The choice of the penalties associated to the objectives can determine the priority quantity within the optimization, and it is similar to the common formulation of the conventional treatment planning problem.

### 2.4 Implementation in matRad: A workflow for cluster dose planning

Dose calculation and cluster dose optimization as described in Section 2.3 was implemented in matRad, an open‐source software for radiotherapy treatment planning with photons and ions.^30^, ^55^ While matRad's PB algorithm was extended to compute cluster dose, the TOPAS MC interface was used together with custom cluster dose scorers, and the optimization routine was extended to allow cluster dose as an additional optimization routine.

For dose calculation, we precalculated general‐purpose (which means not resembling an existing machine) kernel databases for protons, helium, and carbon through MC in TOPAS using OpenTOPAS v.4.0. They contain absorbed dose kernels with the lateral broadening modeled by a double radial Gaussian distribution.

Particle fluence is included, as well as the triple radial Gaussian distributions modeling the lateral fluence broadening for each particle type. The energy of particles used to build the fluence spectra is the one at the beginning of the step* in TOPAS simulations. The sets of particle types considered in our databases are (see also Table 1):

(a) protons: the database for protons includes hydrogen (Z,A)=(1,1), deuterium (Z,A)=(1,2), tritium (Z,A)=(1,3), helium Z=2, and oxygen Z=8.
(b) helium ions: the helium database includes the same particle types as for protons.
(c) carbon ions: the carbon database includes all the particle types with *Z* up to 8, which means the same as the proton and helium database, plus lithium Z=3, beryllium Z=4, boron Z=5, carbon Z=6, nitrogen Z=7, and oxygen Z=8.

**TABLE 1:** Summary of particle types included in fluence kernel database according to primary particle.

Primary particle	Protons		Helium		Carbon	
Particle types included in fluence spectra	*Z*	*A*	*Z*	*A*	*Z*	*A*
	1	$\left\{1,2,3\right\}$	1	$\left\{1,2,3\right\}$	1	$\left\{1,2,3\right\}$
	$\left\{2,8\right\}$	any	$\left\{2,8\right\}$	any	$\left\{2,\text{…},8\right\}$	any

Through post‐processing of the fluence kernels, we included, for each PB, the total cluster dose depth profiles $g^{\left(I_{p}\right)}\left(d\right)$ precomputed in water for all relevant $I_{p}$, $\forall I_{p}\in \left\{F_{k},N_{k}\right|k\in \left[1,\text{…},10\right]$}.

Both cluster dose calculation methods described in Section 2.2.3 were implemented. In the flexible method, matRad reads the particle fluence from the kernel database and interpolates the correspondent $I_{p}$ on the fly, from the MCTS database. In the fast method, precomputed $g^{\left(I_{p}\right)}$ depth profiles are used along with the absorbed dose lateral broadening.

The optimization problem (see Section 2.3) was also implemented: a new set of objectives and constraints, acting directly on cluster dose, was added to matRad. This enables direct steering of cluster dose in radiotherapy treatment planning in matRad.

### 2.5 Treatment planning study on water box and prostate patient

To demonstrate cluster dose treatment planning and validate the kernel algorithm against MC simulations, we compare a set of treatment plans planned with the PB algorithm in matRad to recalculated MC (cluster) dose distributions with matRad's TOPAS interface. To demonstrate the impact and successful steering of cluster dose in optimization, we explore different prescriptions, phantom configurations, and radiation modalities.

For our study we chose to use $F_{5}$ – the preferred $I_{p}$ among $F_{k}$ ∀k=1,…,10, observed for two normoxic cell lines and three radiation modalities^18^, ^56^ – to calculate cluster dose $g^{\left(F_{5}\right)}$. In order to improve calculation time, we used the fast method presented in Section 2.2.3 employing precomputed g‐depth profiles and approximating the lateral broadening of cluster dose with the lateral broadening of absorbed dose. We also added results from calculation of cluster dose using the flexible method relying on fluence‐spectra kernels (Section 3.2) and different $I_{p}$ (Section 3.3).

#### 2.5.1 Box phantom

First, we evaluated cluster dose calculation and optimization on a simple cubic water phantom. The target is a cube of 60mm width, located in the center of a larger water cube of 480mm width that is divided into $160^{3}$ cubic voxels of 3mm edge each. We simulated irradiation with one field, separately exploring a constant absorbed dose SOBP prescription, D=2Gy, and a constant cluster dose SOBP of $g^{\left(F_{5}\right)}=7\;{\mathrm{pg}}^{-1}$ in the target volume, for protons, helium, and carbon ions. There were no constraints applied. For each field, $10^{8}$ primary particles were simulated for MC validation.

#### 2.5.2 Prostate patient

Subsequently, we create several plans with different prescriptions on a prostate patient. We simulated irradiation of target PTV prostate with two opposing lateral fields. For each of the three ions, we again explored an absorbed dose prescription D=2.27Gy, and cluster dose prescription $g^{\left(F_{5}\right)}=7.5\;{\mathrm{pg}}^{-1}$ in the prostate PTV. There were no constraints applied. MC simulations used $10^{8}$ primary particles for each field, for protons and helium, and $10^{7}$ primary particles for each field for carbon.

#### 2.5.3 MC configuration

We use the “Dose To Medium” scorer in TOPAS to calculate the absorbed dose, and we combine the MCTS database with custom scorers to calculate cluster dose. The scorers accumulate the track‐weighted sum of the $I_{p}$: at each simulation step, the $I_{p}^{c}$ value is interpolated on‐the‐fly based on the particle class c, where the particle type and pre‐step kinetic energy are used. This approach aligns with the default fluence filters implemented in TOPAS. Neutrons and particles with zero energy are excluded from the cluster dose scoring.

#### 2.5.4 Comparative analysis

Dose and cluster dose distributions are compared using exemplary isocenter slices, depth profiles, and γ‐analyses using a 2%/2mm distance‐to‐agreement criterion and a threshold of 10% of the maximum dose. For the prostate patient, we also compute dose and cluster dose volume histograms (DVHs). All metrics are computed with matRad.

## 3 RESULTS

### 3.1 Water box phantom: Dose SOBP versus cluster dose SOBP

**FIGURE 1:**
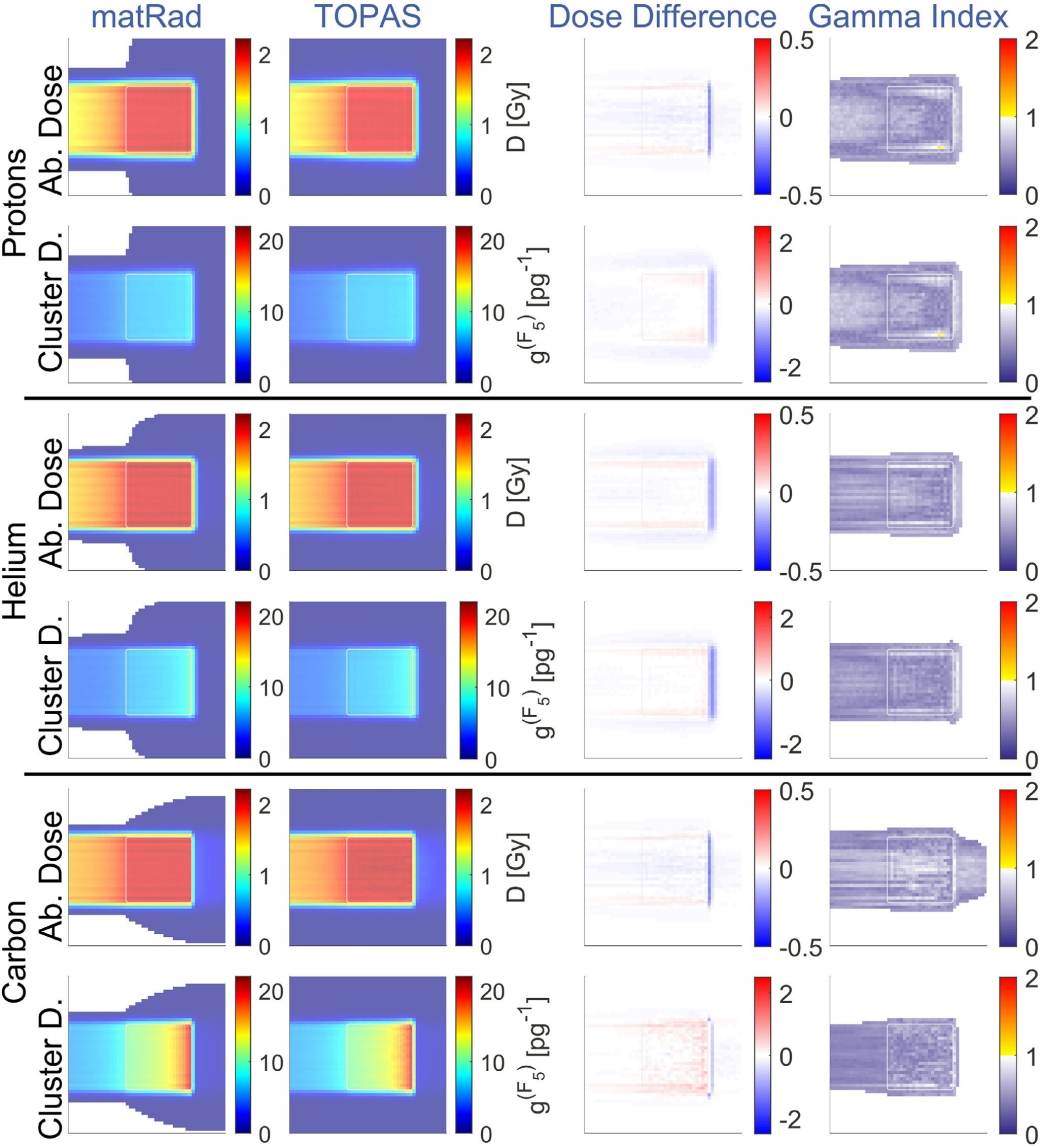
Absorbed dose and cluster dose $g^{\left(F_{5}\right)}$ distributions obtained with a prescribed absorbed dose SOBP, with the prescription D=2Gy within the target volume. Each row displays the absorbed dose (or cluster dose) distribution obtained with PB algorithm in matRad in the first panel, the corresponding result from MC in TOPAS in the second panel, the absolute difference of the two distributions is in the third panel and the Gamma Index in the fourth panel. Rows 1 and 2 are for protons, rows 3 and 4 for helium, and rows 5 and 6 for carbon.

Figure 1 shows the dose distributions for the central slice in the resulting treatment plans on the water box phantom with prescribed constant absorbed dose. For each of the three primary ion species we show the absorbed dose SOBP and the correspondent $g^{\left(F_{5}\right)}$ SOBP. We also show the results of the comparison of the PB kernel computation with the TOPAS computation by means of dose differences and γ‐analyses.

**FIGURE 2:**
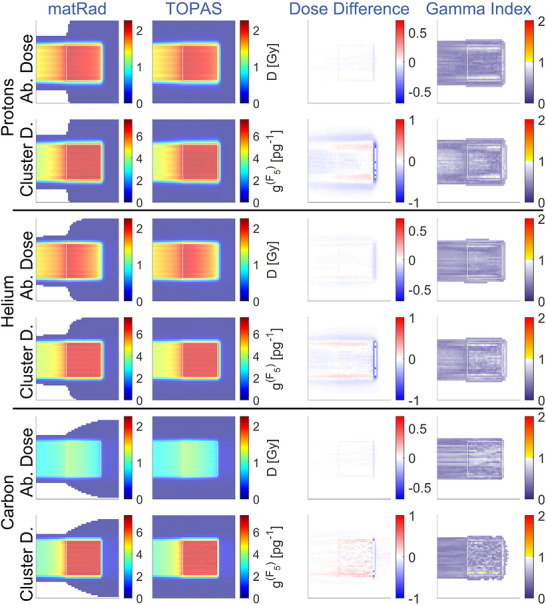
Absorbed dose and cluster dose $g^{\left(F_{5}\right)}$ distributions obtained with a prescribed cluster dose $g^{\left(F_{5}\right)}$ SOBP, with the prescription $g^{\left(F_{5}\right)}=7\;pg^{-1}$ within the target volume. Each row displays the absorbed dose (or cluster dose) distribution obtained with PB algorithm in matRad in the first panel, the corresponding result from MC in TOPAS in the second panel, the absolute difference of the two distributions is in the third panel and the Gamma Index in the fourth panel. Rows 1 and 2 are for protons, rows 3 and 4 for helium, and rows 5 and 6 for carbon.

Figure 2 shows the analogous results for a prescribed constant *cluster dose* $g^{\left(F_{5}\right)}$, that is, “cluster dose SOBP” in the box phantom. The absolute dose and cluster dose differences observed in Figures 1, 2 reveal a systematic lateral scattering inaccuracy most evident for protons, also substantiated by voxels outside of the 3%/3mm gamma criteria in the computed γ distributions. Since differences are consistent between cluster dose calculations and dose calculations, the differences are attributed to a systematic error in lateral scattering. The γ passing rates are all presented in Table 2.

**TABLE 2:** Gamma analysis reflecting the comparison of RTP on a cubic water phantom with matRad versus TOPAS recalculation. Two different prescriptions are applied for each irradiation mode: the first is a prescribed constant absorbed dose SOBP, and the second is a prescribed constant cluster dose SOBP. For each of the six RTP we compare two quantities, that is cluster dose $g^{\left(F_{5}\right)}$ and absorbed dose distributions. We list the GPR obtained with 2%/2mm and 3%/3mm distance‐to‐agreement criterion and threshold of 10% of the maximum dose.

	protons				helium				carbon			
Gamma criteria	Ab. dose SOBP		Cluster dose SOBP		Ab. dose SOBP		Cluster dose SOBP		Ab. dose SOBP		Cluster dose SOBP	
	D GPR	$g^{\left(F_{5}\right)}$ GPR	D GPR	$g^{\left(F_{5}\right)}$ GPR	D GPR	$g^{\left(F_{5}\right)}$ GPR	D GPR	$g^{\left(F_{5}\right)}$ GPR	D GPR	$g^{\left(F_{5}\right)}$ GPR	D GPR	$g^{\left(F_{5}\right)}$ GPR
2mm/2%	96.7%	97.8%	97.2%	97.5%	98.2%	98.6%	99.4%	98.2%	98.6%	99.5%	98.9%	98.9%
3mm/3%	99.1%	99.6%	99.5%	99.4%	99.7%	99.9%	99.9%	99.8%	100.0%	100.0%	100.0%	99.8%

We can identify regions where the γ‐analysis is outside of the criteria inside the target, near the target boundaries and outside the target. But as the gamma passing rates demonstrate, the number of such voxels is small compared to the total. In all the plans on the box phantom, the Gamma Passing Rate values for absorbed Dose are greater than 96.7%, while the values calculated on cluster dose distributions are greater than 97.5%. The GPRs of cluster dose appear to be comparable to the ones of absorbed dose.

**FIGURE 3:**
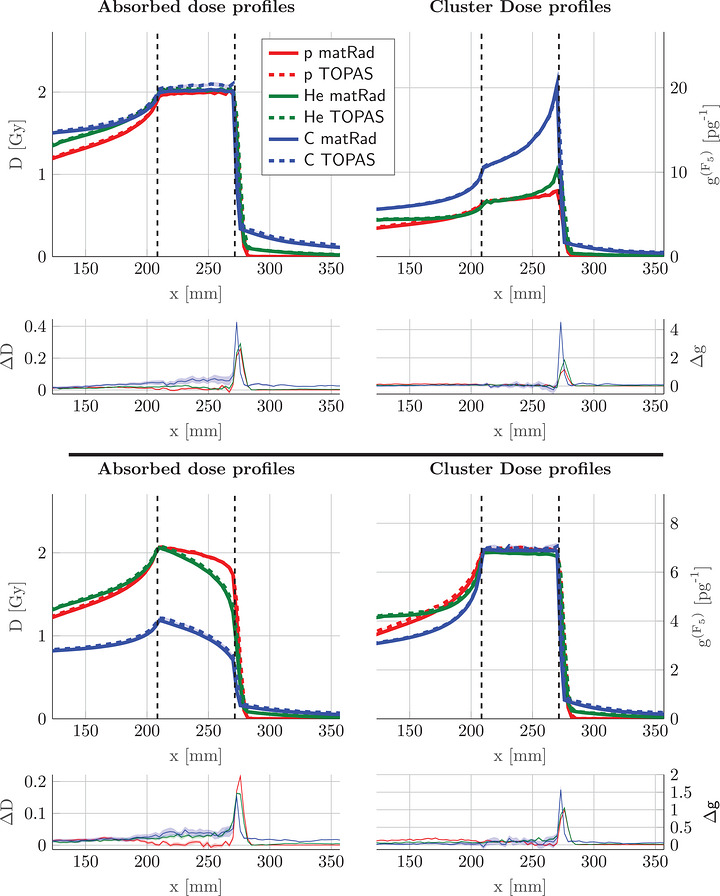
Dose profiles (left column) and cluster dose $g^{\left(F_{5}\right)}$ profiles (right column) for protons (red), helium (green), and carbon ions (blue). The results obtained with PB in matRad (solid line) are plotted against the results obtained with MC in TOPAS (dashed line). Results for absorbed dose SOBP optimization (first row) are separated from cluster dose SOBP optimization (second row). Below each profile plot the absolute dose difference profiles and error (shaded area) are shown.

Figure 3 displays the profiles in depth along the central axis for absorbed dose and $g^{\left(F_{5}\right)}$ from the slice distributions in Figures 1, 2. It also presents the profiles of the absolute dose differences between MC and PB, along with the corresponding standard deviation. The largest differences systematically occur at the distal edge of the field, which corresponds to high‐gradient regions in the dose distributions.

The results in Figures 1, 2, 3 confirm the expected behavior of cluster dose in depth. When simulating a constant dose SOBP, the cluster dose increases toward the distal edge. The magnitude and steepness of this increase depends on the used primary particle, as carbon ions create ionization clusters with a much higher frequency than protons. When optimizing a constant cluster dose SOBP, the physical dose needs to be reduced toward the distal edge.

In the next section we also quantify the expected improvement in cluster dose calculation obtained using the flexible method, that is taking into account the lateral fluence distributions for each primary and secondary particle type (see Section 3.2).

### 3.2 Flexible cluster dose calculation

**FIGURE 4:**
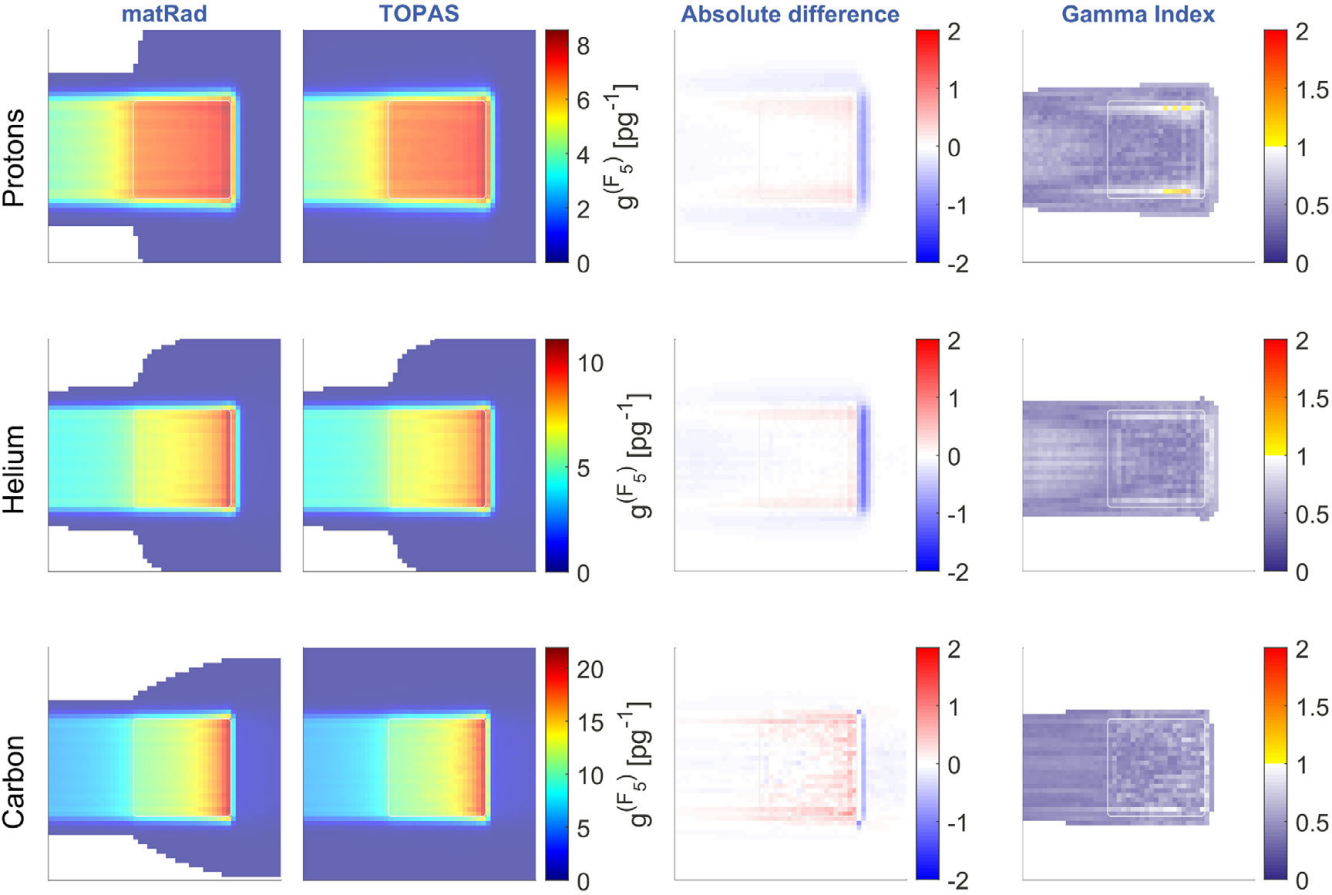
Cluster dose $g^{\left(F_{5}\right)}$ calculated for an absorbed dose SOBP in the box phantom using the fluence kernel spectra in matRad (first column) is compared to the TOPAS recalculation (second column). Absolute difference (third column) and Gamma Index (fourth column) are included. First row is for protons, second row is for helium and third row is for carbon.

In this section we show the recalculation of cluster dose using the flexible method described in Section 2.2, employing full fluence kernels spectra. We consider the previously optimized beam fluence from absorbed dose SOBPs in a box phantom (see Figure 1), and recalculate the corresponding cluster dose $g^{\left(F_{5}\right)}$. Results are displayed in Figure 4, where we chose the same dose slices as in Figure 1 for comparison.

The absolute differences in the cluster dose slices obtained using the flexible method are comparable to the ones obtained with the fast cluster dose calculation displayed in Section 3.1.

In particular, we obtain GPR for $g^{\left(F5\right)}$ of 98.9%, 99.9%, and 100.0% for protons, helium, and carbon ions, respectively. No significant improvement is observed.

### 3.3 Calculation of cluster dose of different $I_{p}$

**FIGURE 5:**
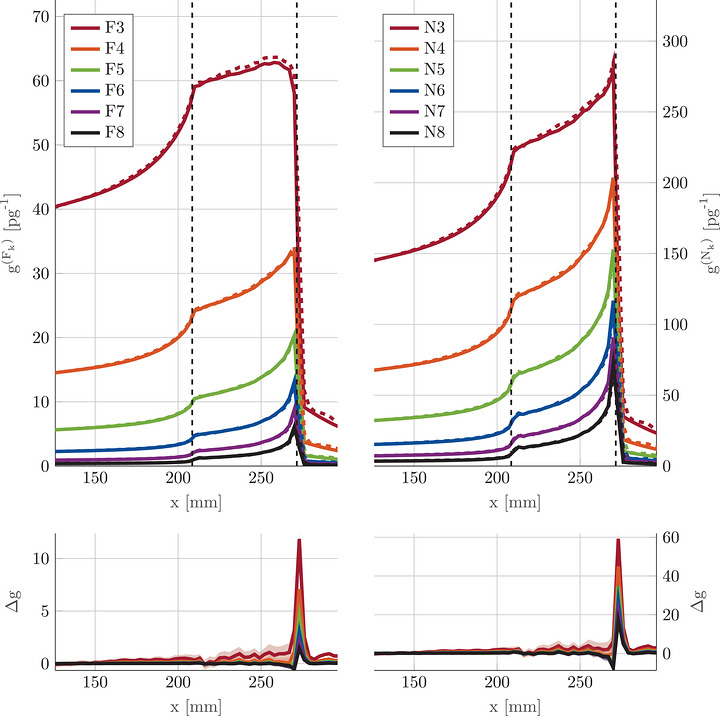
Cluster dose profiles determined by the carbon absorbed dose SOBP, for different $F_{k}$ (left panel) and $N_{k}$ (right panel), computed with matRad's PB algorithms (solid line) and TOPAS (dashed line). Below each profile plot the absolute dose difference profiles and error (shaded area) are shown.

Figure 5 displays superimposed profiles of $g^{\left(F_{k}\right)}$, $g^{\left(N_{k}\right)}$ for each k={3,…,8}, which were produced by the carbon absorbed dose SOBP within a box phantom, calculated with the technique used in Section 3.1, that is the *fast* method for cluster dose calculation.

The results show a consistent expected increase of cluster dose toward the distal edge. Cluster dose values as well as cluster frequencies decrease for larger k, and for all k we observe $N_{k}>F_{k}$ which both follow by definition of the $I_{p}$. The ratio between the peak value and the value at the target entrance is larger for larger k, consistent with the expectation of larger ionization clusters concentrating in the distal edge of the SOBP. All profiles exhibit good agreement between PB calculations and MC simulation.

### 3.4 Prostate patient: Absorbed dose versus cluster dose optimization

**FIGURE 6:**
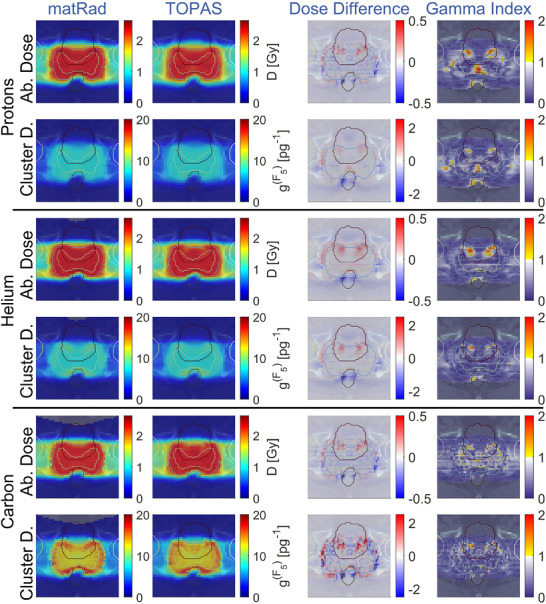
Absorbed dose and cluster dose $g^{\left(F_{5}\right)}$ distributions obtained with a prescribed absorbed dose 2.27Gy in the PTV prostate. Each row displays the absorbed dose (or cluster dose) distribution obtained with PB algorithm in matRad in the first panel, the corresponding result from MC in TOPAS in the second panel, the absolute difference of the two distributions is in the third panel and the Gamma Index in the fourth panel. Rows 1 and 2 show protons, rows 3 and 4 helium, and rows 5 and 6 carbon ions.

The dose and $g^{\left(F_{5}\right)}$ slice distributions obtained with absorbed dose optimization on the PTV prostate are displayed in Figure 6. Again, we compare the pencil‐beam results to the MC results by means of absolute difference and γ‐analysis.

The absorbed dose optimization in the PTV prostate (Figure 6) demonstrates that protons show the most homogeneous $g^{\left(F_{5}\right)}$ within the target volume, if compared to helium and carbon (see also the DVHs in Figures 8, 9, and 10). This is consistent with the $g^{\left(F_{5}\right)}$ profiles previously shown for the absorbed dose SOBP in the box phantom (see Figure 3).

**FIGURE 7:**
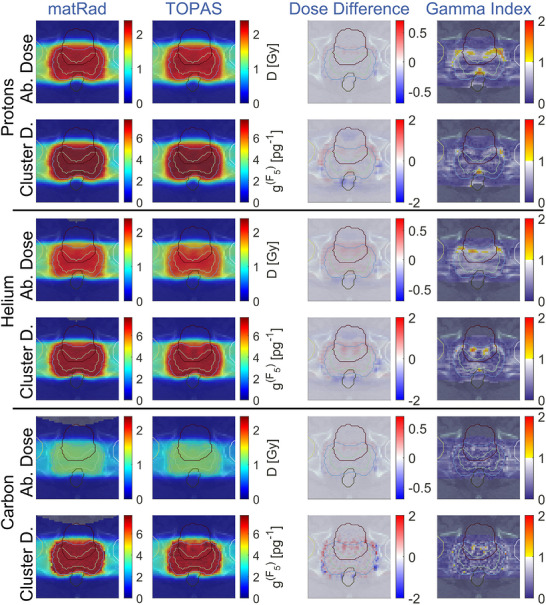
Absorbed dose and cluster dose $g^{\left(F_{5}\right)}$ distributions obtained with a prescribed cluster dose $g^{\left(F_{5}\right)}=7.5\;pg^{-1}$ in the PTV prostate. Each row displays the absorbed dose (or cluster dose) distribution obtained with pencil‐beam algorithm in matRad in the first panel, the corresponding result from MC in TOPAS in the second panel, the absolute difference of the two distributions is in the third panel and the Gamma Index in the fourth panel. Rows 1 and 2 are for protons, rows 3 and 4 for helium, and rows 5 and 6 for carbon.

**FIGURE 8:**
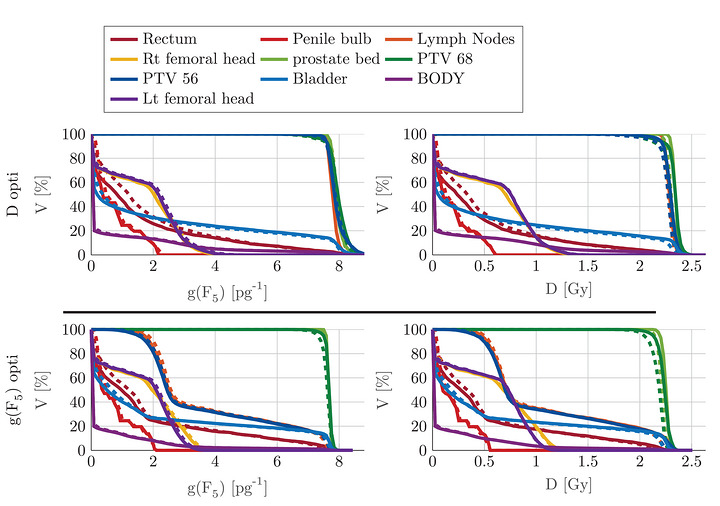
DVH for protons prostate plans, comparing matRad PB (solid) versus TOPAS (dashed). First row shows the results from absorbed dose optimization in the PTV. Second row shows the corresponding results for cluster dose $g^{\left(F_{5}\right)}$ optimization.

**FIGURE 9:**
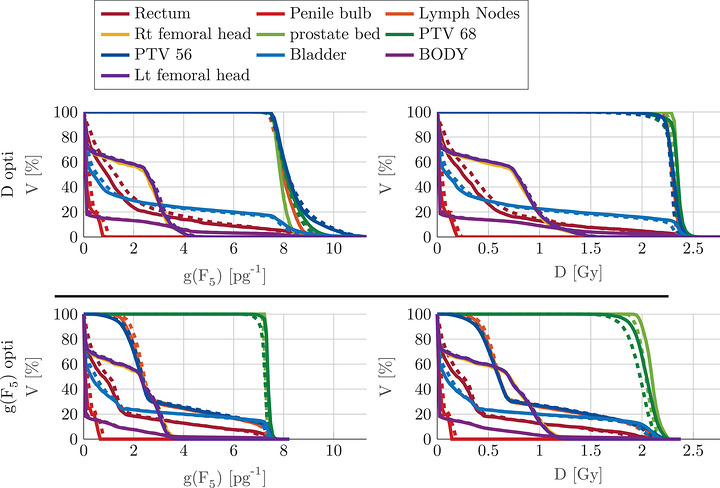
Dose volume histogram for helium prostate plans, comparing matRad PB (solid) versus TOPAS (dashed). First row shows the results from absorbed dose optimization in the PTV. Second row shows the corresponding results for cluster dose $g^{\left(F_{5}\right)}$ optimization.

**FIGURE 10:**
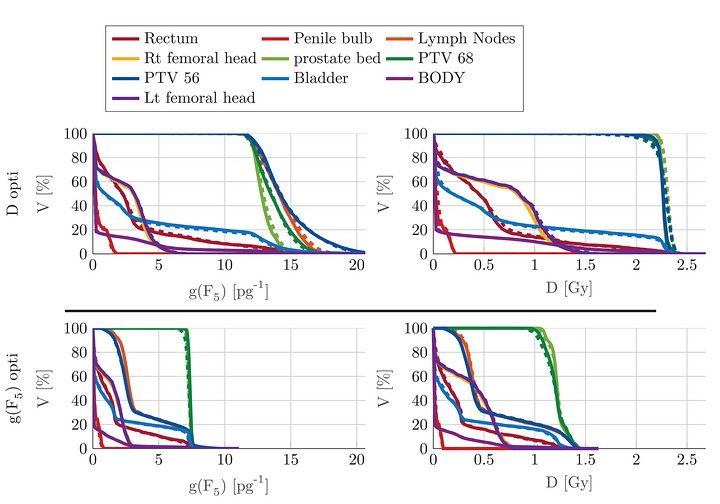
DVH for carbon prostate plans, comparing matRad PB (solid) versus TOPAS (dashed). First row shows the results from absorbed dose optimization in the PTV. Second row shows the corresponding results for cluster dose $g^{\left(F_{5}\right)}$ optimization.

We also applied our tool for prescribing a constant cluster dose level in the PTV prostate. While the prescription of constant cluster dose is based on the expected same biological effect for same $I_{p}$ and same cluster dose level (see Ref. 18), the appropriate values for cluster dose levels and constraints are still to be explored. But in this study, in order to choose a cluster dose level that is also reasonably consistent with the absorbed dose levels prescribed in clinics, we extrapolated the cluster dose level from a proton plan with prescribed constant dose in the PTV, assuming a constant biological effectiveness of RBE = 1.1. Thus cluster dose of $g^{\left(F_{5}\right)}=7.5\;{\mathrm{pg}}^{-1}$ in the PTV prostate was used as a reasonable prescription to optimize the corresponding cluster dose plans for protons, helium, and carbon ions.

The corresponding dose and $g^{\left(F_{5}\right)}$ distributions are displayed in Figure 7. From the prostate plans results we learn that a prescribed constant absorbed dose in the PTV determines increasing cluster dose in the distal edges, and thus peaks of cluster dose at the outer target boundary. This behavior is similar to the box phantom results.

The (cluster) dose difference maps show the largest differences appearing near to the target's outer boundary, that is, in regions with high dose gradients. We can also observe that such boundary regions are characterized by a systematic dose overestimation in the regions intersecting the bladder, and dose underestimation in the regions intersecting the rectum.

The relevant regions where the γ‐analysis is outside the criteria are identified in the proximity of high dose gradients, as for the absolute differences. Other failure regions, visible in the PTV in carbon plans may also stem from lack of sufficient statistics of the MC simulation. Considering the total passing rates in Table 3, we observe values of GPR greater than 92.7% for dose and 94.3% for cluster dose.

**TABLE 3:** Gamma analysis reflecting the comparison of RTP on a prostate patient with matRad versus TOPAS recalculation. For each irradiation mode we compare the two different optimizations, or plans, and for each of them we compare two quantities, that is cluster dose $g^{\left(F_{5}\right)}$ and absorbed dose distributions.

	protons				helium				carbon			
Gamma criteria	Ab. dose optimization		Cluster dose optimization		Ab. dose optimization		Cluster dose optimization		Ab. dose optimization		Cluster dose optimization	
	D GPR	$g^{\left(F_{5}\right)}$ GPR	D GPR	$g^{\left(F_{5}\right)}$ GPR	D GPR	$g^{\left(F_{5}\right)}$ GPR	D GPR	$g^{\left(F_{5}\right)}$ GPR	D GPR	$g^{\left(F_{5}\right)}$ GPR	D GPR	$g^{\left(F_{5}\right)}$ GPR
2mm/2%	94.1%	94.6%	95.3%	96.7%	92.9%	94.3%	94.2%	94.8%	92.7%	96.3%	98.7%	97.3%
3mm/3%	98.4%	99.0%	98.7%	99.5%	97.6%	98.6%	98.4%	98.7%	98.9%	99.1%	99.8%	99.5%

Figure 8 shows the DVHs from proton plans. First, it is possible to see that optimizing on absorbed dose determines a rather homogeneous cluster dose and vice versa.

In PTV, the matRad PB (cluster) dose overestimates the TOPAS MC results: the $D_{95}$ for PTV68 differs by maximum 4.3%. Considering the analogous quality factor for cluster dose, that we can call $G_{95}$ (minimum cluster dose received by 95% of target volume), we obtain a maximum deviation of 2.7%, that is smaller if compared to the absorbed dose deviation. Regarding the maximum deviations in the mean doses in the PTV, for mean absorbed dose is 2.6% and for mean cluster dose is 0.9%. Regarding the OARs, in the bladder the mean dose differs by maximum 2.0%, while the mean cluster dose is underestimated by maximum −2.3%. In the Rectum, the mean dose is underestimated by maximum −11.3% (with mean values of 0.5Gy), and similarly the mean cluster dose by maximum −13.9%.

Figure 9 shows the DVHs from helium plans. In PTVs, $D_{95}$ differs by maximum 6.1%, and $G_{95}$ differs by maximum 4.2%. The mean dose differs by maximum 3.6%, and the mean cluster dose differs by maximum 2.1%. In OARs, the maximum differences in mean doses are visible in Rectum, with −11.1% for the mean absorbed dose (with mean values of 0.4‐0.5Gy) and −14.2% for mean cluster dose. In the bladder, the mean dose differs by maximum 0.5% and the mean cluster dose differs by maximum −2.2%.

Figure 10 shows the DVHs from carbon plans. In PTV68, $D_{95}$ differs by maximum 4.4%, and $G_{95}$ analogously differs by maximum 3.9%. Comparing mean values in PTV68, the maximum deviations are 0.7% for mean absorbed dose, and 1.5% for mean cluster dose. In OARs, the maximum deviations in the mean dose is −2.4% observed in the Rectum. Similarly, the maximum deviation in the mean cluster dose is −2.2% in the Rectum. In the bladder, the mean dose differs by maximum −1.6%, while the mean cluster dose differs by maximum 4.2%.

### 3.5 Comparison of calculation times

The rationale of this work is to enable fast and direct cluster dose calculation using PB algorithm, since full transport MC is currently unfeasible due to its high computational cost. We presented and validated two methods for cluster dose calculation using PB algorithm, namely the *fast* and the *flexible* methods, as already described in Section 2.2.3.

In this section, we provide the computation times for both methods. Table 4 reports the calculation times for the influence matrix across the three radiation modalities explored and the different phantoms. This refers to the stage before fluence optimization, as the latter does not depend on the method used to calculate the $D_{\mathrm{ij}}$ and $G_{\mathrm{ij}}$. As shown in the table, in general, the flexible method leads to an increase in the calculation times of about one order of magnitude. This increase is more pronounced for carbon, as a larger set of particle types is considered in the kernel database.

**TABLE 4:** Calculation times (in seconds) of dose influence matrix for fast and flexible methods. Different radiation modalities and phantom geometries are included.

Modality	Case	Fast [s]	Flexible [s]
Protons	Box phantom	28	254
	prostate	151	1203
Helium	Box phantom	27	254
	prostate	201	1438
Carbon	Box phantom	24	576
	prostate	114	1719

## 4 DISCUSSION

### 4.1 Summary

We demonstrated a workflow for inclusion and direct optimization of nanodosimetric ID in RTP with protons, helium and carbon ions. It employs an extended version of PB algorithm in matRad, that relies on precomputed particle fluence kernels in water to calculate the PB cluster dose. We provided two methods for the cluster dose PB calculation, one faster with a higher degree of approximation, the other slower and more flexible, which uses the full fluence spectra in the kernel data.

We initially validated our tool against MC simulations in TOPAS by exploring different planning configurations, varying patient geometries, radiation modalities, and prescriptions. The validation used primarily $F_{5}$, that is the preferred $I_{p}$ for the normoxic T1 and A549 cell lines.^18^ Other $I_{p}$, namely $F_{3}$ – $F_{8}$ and $N_{3}$ – $N_{8}$, were briefly validated on the box phantom only.

The discrepancies observed in cluster dose, both in a box phantom and a prostate phantom, were comparable to those observed for absorbed dose, with no noticeable improvement in agreement when using the full fluence spectra kernel algorithm instead of the aggregated kernel approach. Overall, the results confirm expectations: discrepancies with MC calculations are primarily driven by limitations in the modeling of lateral scattering rather by definition of cluster dose or the fluence spectra‐based calculation framework itself.

Accurate modeling of lateral particle fluence therefore represents the key requirement for reliable cluster dose planning. For this reason, we compared a fast algorithm – formally analogous to the commonly used PB calculations of biological dose and RBE – with a more detailed modeling of single fluence kernels for different particle types.

This also suggests that adopting advanced PB strategies to improve lateral scattering model, such as subsampling approaches, could further enhance the reliability of cluster dose calculation with comparable accuracy benefits as they would bring to, for example, RBE‐weighted dose calculations.

We explored deriving cluster‐dose prescriptions from conventional proton plans, assuming roughly constant radiobiological effectiveness within the target. Applying these prescriptions to heavier ions led to the expected behavior: lower physical doses are required due to higher frequency of produced nanoscopic ionization clusters, as observed in both the cubic and the prostate phantoms, in line with prior findings on RBE‐weighted dose^57^, ^58^ and LET distributions.^59^, ^60^

### 4.2 Scope and limitations

The presented approach to cluster dose treatment planning and its MC validation may pave the way for the extension to other nanodosimetric quantities and its integration and comparison with conventional RBE‐weighted dose optimization.

The presented study was limited to cluster dose based on $I_{p}$ defined as $F_{k}$ or $N_{k}$, yet our approach would directly allow extension to other linear operators on the frequency distributions. In addition, since different cell lines under varying oxygenation conditions are expected to also have different preferred $I_{p}$,^18^ choosing to calculate different $I_{p}$ by voxel or structure (similar to α/β ratios) can further be enabled with our approach.

The integration and comparison with RBE‐weighted planning could be approached initially with dual planning approaches, where cluster dose or voxel‐averaged $I_{p}$ steer a clinically acceptable RBE‐weighted planning approach toward a favorable distribution of ionization clusters, already proposed by previous studies.^24^, ^61^ Here, it would be natural to connect to existing work on LET‐driven planning^11^, ^12^, ^13^ and multi‐ion therapy seeking homogeneous dose‐weighted LET distributions.^62^

Although the connection to LET optimization seems natural, it is important to reconsider the analogy “LET relates to dose as $I_{p}$ relates to cluster dose” from Section 1. Cluster dose is a fluence‐dependent quantity expressing the actual number of ionization clusters per unit mass. On the other hand, LET is a dose‐averaged quantity usually included as an additional constraint in standard optimization algorithms, which allows to steer local radiation quality while maintaining the prescribed dose level. This suggests that the averaged $I_{p}$ could serve as a surrogate of LET to improve cluster dose‐optimized plans. Future work could explore steering an averaged $I_{p}$ in optimization, alongside LET‐, RBE‐ or cluster dose‐weighted quantities. Different averaging definitions may be considered, with a fluence‐weighted $I_{p}$ likely being most appropriate under the assumption that clustered ionization events contribute similarly to DNA damage regardless of particle type or energy. Hence, not only are the two quantities conceptually different, but defining the appropriate analogous objectives is a nontrivial matter. We therefore leave this to future studies, which may take advantage of the opportunities opened by the framework presented in this work.

At this point, it is important to acknowledge that the nanodosimetric model used here is not a biological model, but describes novel physical quantities to be used on the macroscopic treatment planning level. Validating the full biological cluster dose response relationship in the context of RTP lies beyond the scope of this work. The relevance of the model is to provide a new, more physically detailed description of radiation quality that can improve consistency across different ion species. Still, such a stronger biological link could facilitate the prediction of DNA damage at the treatment planning scale, when additional parameters, such as nuclear size, repair mechanisms, and chromosomal aberrations, are included and condensed into a stochastic damage model relying on macroscopic parameters. Complementing this with further radiochemical effects like, for example, the probability of radical production, could potentially allow to define a full‐fledged biological model directly on cluster dose to enable direct biological cluster dose planning.

Validity of such future planning studies relies on the validity of the underlying nanodosimetric planning framework. While this work validated the cluster dose planning approach on the level of the macroscopic calculations relying on a MCTS database, it will propagate any systematic errors from the underlying track‐structure simulations, thus questioning the extent to which condensed history MC can serve as a valid “gold standard”.

For the MC simulations within this work, we relied on a condensed‐history (CH) approach, where cluster dose is scored by look‐up from an ID database precomputed from track‐structure (TS) simulations. The principal feasibility and consistency of such an approach was previously validated by Ramos–Méndez et al.^17^, where the CH approach with ID lookup was compared against track structure simulations initialized with the corresponding local phase space. While not directly transferable to cluster dose employed in this work, since it is not an averaged local beam property, this previous validation forms a reasonable basis for the database‐centered approach in CH simulations. Still, systematic uncertainties arising from the track structure simulation geometry and database design will propagate to the CH simulation result.

Ortiz et al.^52^ analyzed the impact of refinements to the TS geometry and CH lookup on cluster dose calculation, demonstrating variations of cluster dose based on $F_{5}$ typically within 20%. Exceptionally, protons show larger variations of up to a factor three in their work, which reduces when smaller clusters are considered. This further demonstrates that cluster dose computation is highly susceptible to systematic uncertainties in the underlying IP database track structure simulation, especially when comparing to the much more stable computation of conventional absorbed dose, while at the same time showing a stronger association with radiobiological endpoints such as clonogenic cell survival. Thus, even if works like Ramos–Méndez et al.^17^ or this work itself observe consistency between two computational approaches, this underlying uncertainty should always be questioned before considering the computed cluster dose as absolute.

Furthermore, previous studies have shown that different MCTS codes may exhibit substantial discrepancies in the calculation of nanodosimetric quantities. These differences arise from several factors, such as different underlying physics models, the availability of cross section data, and interpolation methods.^63^, ^64^

Some of these discrepancies between MCTS approaches highlight the need for a proper characterization of cross section data for secondary, low‐energy electrons.^65^ Beyond cross‐section uncertainties, another MCTS‐related critical aspect regarding secondary electrons is ensuring consistency in their “count” between MCTS simulations and macroscopic calculation approaches.^52^ The present work includes electrons implicitly considered in the MCTS database, meaning that electrons within a range of approximately 100nm from the primary track are accounted for. This, however, excludes δ‐rays with a larger range, which could lead to an underestimation of the cluster dose as their ID is not explicitly considered. Such future changes to the inclusion of δ‐rays would not compromise the validity of our approach as long as handling of δ‐rays is consistent between the MC simulation and the PB kernels. This would become even more relevant in case the formalism should also be applied to photons, for example to benchmark biological effect models using secondary electron track simulations. In this context, evaluating track overlaps and higher‐order clusters may become increasingly relevant.

Further, besides the aforementioned typical systematic errors in PB computations and their kernel formulation, the kernel computations in water ignore differences of ID in the various media encountered within the human body. However, this limitation translates to the availability of MCTS data and is also mitigated by the fact that water still makes up the predominant medium in the cell nucleus.

The herein presented approach may, however, facilitate future studies on how these systematic uncertainties in TS computation propagate to macroscopic nanodosimetric treatment planning. For example, the MCTS database could be easily populated with custom frequency distributions, for example subject to different simulation parameters or geometries,^52^ or be fed into independent macroscopic cluster dose scorers originating from other MCTS codes and secondary electron integration approaches.

Regarding experimental validation, the direct measurement of cluster dose under clinical conditions remains an open challenge. The currently available nanodosimetric detectors are based on millimeter‐sized sensitive volumes operating for gaseous media, and they exhibit different responses.^21^ While awaiting further advancements in nanodosimeter technology, indirect validation strategies may be considered for experimental purposes. One possibility is to measure particle fluence, which constitutes one of the components of cluster dose. Alternatively, when the source characteristics are known, the absorbed dose can be predicted in cluster dose‐optimized plans and, at the same time, it can be measured using standard quality assurance procedures. In both cases, the $I_{p}$ remains a predictive quantity derived from modeling. Here, the generation of experimental dose distributions exhibiting dedicated cluster dose characteristics, like homogeneous cluster dose SOBPs, may be supported by our approach.

## 5 CONCLUSION

This work presents a MC validated cluster dose treatment planning framework within matRad for protons, helium and carbon ions. Using prototypical plans on a box phantom and a prostate patient showed that established pencil‐beam algorithms for cluster dose calculation yield typical PB accuracy compared to MC simulations in TOPAS.

Consequently, our framework provides a fast and valid cluster dose planning for nanodosimetric research, while retaining flexibility for custom track structure databases and/or other ID parameters.

Whether fully cluster dose‐based clinical planning will be achievable in the future will depend on future developments such as (1) biological models that incorporate nanodosimetric quantities, (2) validation and benchmarking of track‐structure simulations, (3) stronger evidence supporting selection of appropriate $I_{p}$ across different cell lines and oxygenation conditions, and (4) accurate experimental validation of radiation effect in cluster dose treatment plans. By being able to provide clinically deliverable cluster dose treatment plans, our approach may serve to support such studies.

## CONFLICT OF INTEREST STATEMENT

The authors declare no conflicts of interest.

## Footnotes

* The option to bin fluence by the initial energy in a MC step is indicated with “Prestep” in TOPAS.

## APPENDIX A FORMAL DETAILS OF THE FRAMEWORK

### A.1 Triple Gaussian model for fluence lateral broadening

Within a monoenergetic beam, and for a specific primary or secondary particle, we model the lateral broadening of particle fluence with a triple radial Gaussian model:

(A1)
$$G_{\sigma }\left(\rho \right)\equiv G\left(0,\sigma ,\rho \right)=\frac{e^{-\rho^{2}/2\sigma^{2}}}{2\pi \sigma^{2}},$$

(A2)
$$\int_{0}^{\infty }G_{\sigma }\left(\rho \right)2\pi \rho \;d\rho =1,$$

(A3)
$$\begin{array}{cc} L(\rho ) & \equiv \left(1-\omega_{2}-\omega_{3}\right)\cdot G_{\sigma_{1}}\left(\rho \right)+\omega_{2}\cdot G_{\sigma_{2}}\left(\rho \right)\\ & \;+\omega_{3}\cdot G_{\sigma_{3}}\left(\rho \right), \end{array}$$

Here, $G_{\sigma }\left(\rho \right)$ is the single radial Gaussian distribution, and L(ρ) indicates the normalized lateral fluence distribution. For each (Z,A) the depth‐dependent set $\left\{\sigma_{1}\left(d\right),\sigma_{2}\left(d\right),\sigma_{3}\left(d\right),\omega_{2}\left(d\right),\omega_{3}\left(d\right)\right\}$ is stored, obtained by fitting particle fluence from MC simulations of PBs in water.

### A.2 Flexible cluster dose calculation with spectral fluence kernels

The flexible method relies on precomputed fluence kernels and on‐the‐fly interpolation of the $I_{p}$.

We refer again to Equation 5, which expresses the contribution of a particle type s to cluster dose at a given depth. In order to express the cluster dose contribution within a bin volume at a radial distance r from the beam axis, we must multiply $g^{\left(I_{p}\right)\left[s\right]}\left(d_{j}\right)$ by the correspondent lateral scattering. If $L_{d_{j}}^{\left[s\right]}\left(r_{k}\right)$ represents the lateral broadening of fluence for particle type s at depth bin dj and radial distance bin $r_{k}$, we approximate the total cluster dose within the same bin volume as:

(A4)
$$g^{\left(I_{p}\right)}\left(d_{j},r_{k}\right)=\sum_{s}g^{\left(I_{p}\right)\left[s\right]}\left(d_{j},r_{k}\right)\approx \sum_{s}L_{d_{j}}^{\left[s\right]}\left(r_{k}\right)g^{\left(I_{p}\right)\left[s\right]}\left(d_{j}\right)\;.$$

Here we approximate the lateral broadening of cluster dose using that of the fluence, assuming depth dependence and neglecting the effects of a change in lateral energy distributions. This choice is motivated by our aim to use the lateral broadening separated by particle type, which is directly available for fluence from MC simulations without any post‐processing modifications.

### A.3 Fast cluster dose calculation

The fast method uses precomputed total cluster dose kernels and the lateral broadening of absorbed dose.

The total cluster dose at depth bin $d_{j}$ and radial distance $r_{k}$ is then expressed as:

(A5)
$$g^{\left(I_{p}\right)}\left(d_{j},r_{k}\right)\approx g^{\left(I_{p}\right)}\left(d_{j}\right)\;L_{d_{j}}^{dose}\left(r_{k}\right)\;.$$

Here, $L_{d_{j}}^{dose}\left(r_{k}\right)$ indicates the absorbed dose lateral broadening, modeled as a single or double Gaussian.
